# A Medical Astronomer

**Published:** 1860-04

**Authors:** 


— At the meeting of the Academy of Sciences in Paris, on December 26th, 1859, M. Leverrier made the following interesting communication: Dr. Lescarbault, a medical man in busy practice at Orgères, in the department of the Eure-et-Loire, is also a zealous astronomer, and a man who supplies by his ingenuity the deficiency of the means which he possesses for prosecuting his favorite science. In March last, M. Lescarbault observed the passage over the sun's disc of a planet within the orbit of Mercury, and he communicated the fact to M. Leverrier, who had noticed certain perturbations in the motion of Mercury, that, in his opinion, could only be explained by the presence of another planet. This was in September last; and thereupon M. Leverrier visited him, together with M. Vallée, and had been enabled to confirm the title of M. Lescarbault to the discovery. The correctness of the results obtained by him was the more remarkable on account of the paucity of his instruments. On arriving at Orgères, M. Leverrier found a regular observatory, with instruments chiefly contrived by the doctor himself, whose finances were limited. Not having a chronometer, he had made himself a pendulum, striking seconds, by means of an ivory ball and a bit of string. Notwithstanding the clumsiness of his apparatus, the calculations of M. Lescarbault varied less from those of M. Leverrier than those of the most eminent astronomers sometimes do from each other. For want of paper, Dr. Lescarbault had generally written down his observations with charcoal on a deal-board; which, with the doctor's calculations written on it, was presented to the Academy by M. Leverrier.

— Subnitrate of bismuth is, M. Velpeau says, of great service in erythematous affections of the sk n; there does not exist a better astringent or resolvent; it is also precious in affections of the conjunctiva, because it does not irritate.

